# Impact of Weaning from Acute Dialytic Therapy on Outcomes of Chronic Kidney Disease following Urgent-Start Dialysis

**DOI:** 10.1371/journal.pone.0123386

**Published:** 2015-04-09

**Authors:** Yung-Ming Chen, Wen-Yi Li, Vin-Cent Wu, Yi-Cheng Wang, Shang-Jyh Hwang, Shih-Hwa Lin, Kwan-Dun Wu

**Affiliations:** 1 Renal Division, Department of Internal Medicine, National Taiwan University Hospital, Yun-Lin Branch, Yun-Lin, Taiwan; 2 Renal Division, Department of Internal Medicine, National Taiwan University Hospital, College of Medicine, National Taiwan University, Taipei, Taiwan; 3 Division of Nephrology, Department of Internal Medicine, Kaohsiung Medical University Hospital; Faculty of Renal Care, College of Medicine, Kaohsiung Medical University, Kaohsiung, Taiwan; 4 Adjunctive Investigator, Division of Geriatrics and Gerontology, Institute of Population Health Sciences, National Health Research Institutes, Taiwan; 5 Division of Nephrology, Department of Internal Medicine, Tri-Service General Hospital, National Defense Medical Center, Taipei, Taiwan; I2MC INSERM UMR U1048, FRANCE

## Abstract

Discontinuation of acute, unplanned dialysis is always an important therapeutic goal in dialysis-requiring patients with existing chronic kidney disease. Only a limited proportion of patients could be weaned off dialysis and remained dialysis-free. Here we performed a multicenter, observational study to investigate factors associated with successful weaning from acute dialysis, and to explore the potential impact of weaning itself on outcomes of patients with chronic kidney disease following urgent-start dialysis. We recruited 440 chronic kidney disease patients with a baseline estimated glomerular filtration rate <45 ml/min per 1/73 m2, and used propensity score-adjusted Cox regression analysis to measure the effect of weaning from acute dialysis on death during the index hospitalization and death or readmission after discharge. Over 2 years, 64 of 421 (15.2%) patients who survived >1 month died, and 36 (8.6%) were removed from dialysis, with 26 (6.2%) remaining alive and dialysis-free. Logistic regression analysis found that age ≧ 65 years, ischemic acute tubular necrosis, nephrotoxic exposure, urinary obstruction, and higher predialysis estimated glomerular filtration rate and serum hemoglobin were predictors of weaning off dialysis. After adjustment for propensity scores for dialysis weaning, Cox proportional hazards models showed successful weaning from dialysis (adjusted hazard ratio 0.06; 95% confidence interval 0.01 to 0.35), along with a history of hypertension and serum albumin, were independent protectors for early death. Conversely, a history of stroke, peripheral arterial disease and cancer predicted the occurrence of early mortality. In conclusion, this prospective cohort study shows that compared to patients with chronic kidney disease who became end-stage renal disease after acute dialysis, patients who could be weaned off acute dialytic therapy were associated with reduced risk of premature death over a 2-year observation period.

## Introduction

Patients with existing chronic kidney disease (CKD) are susceptible to the development of end-stage renal disease (ESRD) when put on dialysis acutely due to unexpected decline in renal function complicated by uremic urgency [1–3]. In the event that intrinsic kidney function does not recover after treatment, the resultant unplanned start to long-term renal replacement therapy (RRT) can be associated with a high level of morbidity and mortality [4]. Furthermore, the premature initiation of RRT will impose a heavier burden on the tightened healthcare budget in most industrialized countries [5,6], including Taiwan where the incidence and prevalence of treated ESRD is among the highest in the world [7].

A reasonable and effective approach to reduce the rate of new-onset ESRD is to try weaning off acute, unplanned dialytic therapy if appropriate [8]. Nevertheless, unlike patients with isolated acute kidney injury (AKI) with or without acute dialysis, patients with existing CKD after urgent-start dialysis are usually thought to progress to ESRD over a short period of time. Consequently, when these patients are faced with the decision-making of dialysis weaning, chances are that they may not receive sufficient attempts to taper off the dialytic therapy, and only a limited proportion of patients could be weaned off dialysis successfully. In addition, even in patients with isolated AKI who require acute RRT, criteria for discontinuing acute dialysis are still lacking owing to a variety of patient factors and facility limitations [9,10]. Our retrospective analysis had shown that around 10% of dialysis-requiring patients with acute-on-chronic renal failure could be weaned off dialysis and remained dialysis-free without adversely affecting overall survival after an average follow-up of 5 years [11]. However, to our knowledge, there has been no prospective research focusing on the safety and feasibility issues of dialysis weaning in patients with known CKD. Given the worldwide epidemic of CKD and growing burden of ESRD [7,12,13], this multicenter, observational study was conducted to investigate factors associated with successful weaning from acute dialysis, and the impact of weaning itself on outcomes of patients with CKD following urgent-start dialysis over a 2-year observation period.

## Materials and Methods

This prospective observational study recruited patients with stage 3b to 5 CKD [14] who were put on dialysis acutely via a temporary central venous catheter (CVC) between December 2008 and July 2011 from 3 academic medical centers in Taiwan. The study protocol was approved by the Research Ethics Committee of National Taiwan University Hospital (No. 200810041R) and the Institutional Review Board of Tri-Service General Hospital (No. 070-05-114) and Kaohsiung Medical University Hospital (No. 980078). A total of 471 adult patients were approached for participation in this study. After excluding 31 patients who declined the invitation, 440 patients were enrolled. Written informed consent was obtained from all participants before the start of the study. The diagnosis of AKI followed the criteria proposed by the AKI Network [15]. The determination of presumed etiology for acute-on-chronic renal failure was based on the PICARD study group [16]. The diagnosis of comorbidity was documented by clinically relevant history, medical examinations, or pathological reports. The categories of cardiovascular disease included arrhythmia, cerebrovascular infarcts or hemorrhage, congestive heart failure, coronary artery disease, peripheral vascular disease, and valvular heart disease.

### Initiation of and weaning off acute dialysis

Acute dialysis was initiated for fluid overload, hyperkalemia, or other uremic urgency by the treating nephrologists based on criteria established and mandated by the National Health Insurance Administration, Taiwan [17]. Decisions to discontinue dialysis were made based on improving clinical conditions, including tapering of inotropic agents, weaning from mechanical ventilation, increase in urine output, decline in predialysis blood urea nitrogen and creatinine levels. This process was decided on an individual basis by each clinician and did not follow a uniform protocol. Patients who could be weaned off dialysis without redialysis ≧90 days, i.e., the weaners, were followed at outpatient nephrology clinics and cared by CKD multidisciplinary teams, while those who remained dialysis-dependent, i.e., the non-weaners, received in-center hemodialysis or peritoneal dialysis therapy.

### Study Measures and Data Collection

The primary end point was all-cause mortality during the index hospitalization and after discharge, and the secondary end point was overall readmission. Any events of death or hospitalization along with their causes were evaluated and recorded. Surviving patients were censored at the time of transplantation or the end of the observational period. In patients who did not adhere to scheduled clinic visits, the status of their outcomes was confirmed via telephone at 2 years of follow-up. Baseline demographic, clinical, and biochemical characteristics were recorded at enrollment. Baseline eGFR and CKD stage were determined by the Chronic Kidney Disease Epidemiology Collaboration equation [18], using serum creatinine measured or documented in electronic medical records 3 to 6 months prior to initiation of acute dialysis. The size of kidneys was obtained based on the latest renal sonography before acute dialysis. The size of the larger kidney was chosen for analysis.

### Statistical Analyses

All statistical analyses were carried out using PASW Statistics for Windows version 18.0.0 (SPSS Inc., Chicago, IL). The distributional properties were expressed as mean ± standard deviation for numerical data and frequency with percentage (%) for categorical variables, unless otherwise specified. In comparisons between groups, 2-sample t test and Mann-Whitney U test were used for numerical data and Chi-squared test or Fisher’s exact test (if cells had expected counts <5) for categorical variables. Survival analyses of time to overall mortality or readmission were performed using the Kaplan and Meier method and the log-rank test. The observed time started from the point when acute dialysis commenced via a CVC. Multivariate logistic regression analysis and Cox proportional hazards model were conducted to determine independent predictors associated with weaning off acute dialysis, and overall mortality and hospital readmission, respectively. Univariate analysis was first done for each variable between groups, and those (variables) with a p value ≤0.10 or clinical importance were included in the final multivariate model using stepwise forward selection method. To reduce bias in assessing the effects of dialysis weaning, we used a propensity score approach to construct a comparable non-weaner group with balanced characteristics. The propensity score for each patient based on the probability of successful dialysis weaning was then included in the analysis of Cox proportional hazards model. The assumption of proportional hazards was verified with the log-minus-log graphical method. Significant differences were defined as p value less than 0.05.

## Results

### Clinical characteristics and overall outcomes

After initiation of acute dialysis, 19 (4.3%) of 440 patients died within 30 days during the index hospitalization. These patients were excluded for outcome analysis due to insufficient observation period for decision-making of dialysis weaning. 421 (95.7%) patients who survived more than 1 month and adhered to clinic visits after discharge were followed over 2 year. The baseline clinical features are listed in Table 1. During the study period, 36 (8.6%) of 421 patients were successfully weaned from acute dialysis (the weaners), and 385 (91.4%) became ESRD (the non-weaners). Among the weaners, 5 (13.9%) died, 26 (72.2%) remained dialysis-free, and 5 (13.9%) restarted dialysis; whereas of the non-weaners, 59 (15.3%) died, 9 (2.4%) received kidney transplantation, and 317 (82.3%) continued maintenance hemodialysis or peritoneal dialysis. The mean number of readmissions per 100 patient months was 6.5 and the mean hospital days per admission was 12.8 (median 8.4).

**Table 1:** Baseline demographic characteristics and biochemical data in all participants, and between the weaner and non-weaner groups.

	All	Weaners	Non-weaners
No. of patients	421	36	385
Men, no. (%)	240 (57)	18 (50)	222 (58)
Mean age at entry (y)	61.9±16.4	70.4±12.6	61.1±16.5**
≧65 yr, no. (%)	192 (46)	26 (72)	166 (43)**
Primary renal disease, no. (%)			
Diabetes mellitus	185 (44)	15 (42)	170 (44)
Glomerulonephritis	93 (22)	5 (14)	88 (23)
Others	143 (34)	16 (44)	127 (33)
Comorbidity at entry, no. (%)			
Diabetes mellitus	213 (51)	18 (50)	195 (51)
Hypertension	342 (81)	28 (78)	314 (82)
Dyslipidemia	106 (25)	14 (39)	92 (24)*
CAD	69 (16)	12 (33)	57 (15)**
CHF	84 (20)	11 (31)	73 (19)
VHD	25 (6)	1 (3)	24 (6)
Arrhythmia	5 (1)	0 (0)	5 (1)
CVA	39 (9)	6 (17)	33 (7)
PAOD	30 (7)	3 (8)	27 (7)
Cancer	40 (10)	6 (17)	34 (9)
Acute-on-chronic precipitating factor, no. (%)			
Ischemic ATN	23 (6)	4 (11)	19 (5)
Nephrotoxic	27 (6)	5 (14)	22 (6)
Cardiac	157 (37)	10 (28)	147 (38)
Inflammatory/infectious	20 (5)	2 (6)	18 (5)
Hepatic	1 (0)	1 (3)	0 (0)
Obstructive	12 (3)	7 (19)	5 (1)**
Nil^+^	181 (43)	7 (19)	174 (45)**
Renal sonography at entry			
Kidney size, cm	9.5±1.5	10.3±1.3	9.4±1.5**
Baseline CKD stage, n (%)			
Stage 3b	4 (1)	2 (5.6)	2 (0.5)*
Stage 4	34 (8)	12 (33.3)	22 (5.7)**
Stage 5	383 (91)	22 (61.1)	361 (93.8)**
Renal function at acute dialysis			
Blood urea nitrogen, mg/dL	122.4±45.9	106.5±55.6	123.9±44.7**
Creatinine, mg/dL	11.8±5.4	7.4±2.8	12.2±5.4**
eGFR (CKD-EPI), mL/min/1.73 m^2^	4.6±2.5	7.2±3.9	4.3±2.2**
Laboratory data at acute dialysis			
Albumin, g/dL	3.4±0.6	3.3±0.6	3.4±0.6
Hemoglobin, g/dL	8.6±1.8	9.8±2.2	8.5±1.7**
Potassium, mmol/L	4.7±1.1	5.3±1.7	4.7±1.0
Phosphorus, mg/dL	6.9±2.3	6.0±2.1	6.9±2.3*
Calcium, mg/dL	8.0±1.3	8.5±1.6	8.0±1.3

Abbreviations. ATN, acute tubular necrosis; CAD, coronary artery disease; CHF, congestive heart failure; CVA, cerebrovascular accident; eGFR, estimated glomerular filtration rate; PAOD, peripheral arterial occlusive disease; VHD, valvular heart disease. Values are expressed as number (percent) or mean ± standard deviation.

*<0.05;

**<0.01 vs. weaners.

^+^Nil denotes absence of classic acute precipitating factors, and acute dialysis was started due to unexpected occurrences of hyperkalemia, metabolic acidosis, or gastrointestinal symptoms such as nausea/vomiting.

### Predictors of dialysis weaning

The distribution of baseline CKD stages before acute dialysis is shown in Table 1. 38.9% of the weaners had preexisting stage 3b or 4 CKD, and 44% showed reversible precipitating factors such as ischemic acute tubular necrosis (ATN), nephrotoxic exposure or urinary obstruction. By contrast, the baseline CKD stage in the non-weaners was mostly stage 5 (93.8%), and nearly half of them did not have classic precipitating factors prior to acute dialysis (the “Nil” group). The top 3 offending nephrotoxic agents were herbs (n = 18, 1 weaner), nonsteroidal anti-inflammatory drugs (n = 5, 1 weaner), and radiocontrast exposure (n = 4, 3 weaners). The major cardiac conditions were pulmonary edema due to fluid overloads (n = 124, 9 weaners), congestive heart failure (n = 22, 1 weaner), and coronary artery disease (n = 11, all non-weaners). Among those, 11 cases (all non-weaners, 4 with congestive heart failure and 7 with coronary artery disease) were related to radiocontrast exposure. Additional comparisons show that the weaners were older, and had more comorbid illnesses of dyslipidemia and coronary artery disease. They also had a higher eGFR and greater kidney size, lower serum creatinine, blood urea nitrogen and phosphorus, and a higher hemoglobin at start of acute dialysis. Multivariate logistic regression analysis found that age ≧65 years, presence of ischemic ATN, exposure to nephrotoxin, urinary obstruction, higher eGFR, and higher serum hemoglobin were independent predictors of successful weaning from acute dialysis. We applied these variables to calculate the predicted probability of being assigned to dialysis weaning for each patient to minimize the baseline differences between groups. The predicted probability of dialysis weaning = 1/(1 + exp[-(-10.80+1.28 x (age category) + 1.71 x (ischemic ATN) + 1.50 x (nephrotoxic) + 3.10 x (obstructive) + 0.20 x eGFR + 0.36 x hemoglobin)]) (see footnotes below Table 2). The validation test showed an adjusted R^2^ of 0.28, and the area under the receiver operating characteristic value was 0.84. The predicted probability derived from this model was used as a covariate for subsequent Cox regression analysis for time to clinical end points.

**Table 2:** Multivariate logistic regression analysis showing predictors for successful weaning from acute dialysis, constructed to the propensity score.

Covariate	Estimated coefficient	Standard error	p value	Odds ratio	95% CI	
					Lower	Upper
Age category (≧65 vs. <65 yr)	1.28	.49	.010	3.59	1.37	9.44
eGFR (per mL/min/1.73m^2^)	.20	.08	.010	1.22	1.05	1.42
Hemoglobin (per g/dL)	.36	.12	.003	1.43	1.13	1.80
Acute-on-chronic precipitating factor (yes vs. no)						
Ischemic ATN	1.71	.77	.026	5.51	1.23	24.82
Nephrotoxic	1.50	.71	.034	4.46	1.12	17.78
Obstructive	3.10	.92	.001	22.10	3.67	133.18

Abbreviations. ATN, acute tubular necrosis; eGFR, estimated glomerular filtration rate. The multivariate logistic regression model was conditioned on kidney size and other comorbidities including diabetes mellitus, and showed a percentage of concordant pairs = 79.3%, adjusted generalized R^2^ = 0.276, estimated area under the receiver operating characteristic curve = 0.841, and Hosmer and Lemeshow goodness of fit test p = 0.107>0.05. The predicted probability (propensity score) of dialysis weaning = 1/(1 + exp[-(-10.80+1.28 x (age category) + 1.71 x (ischemic ATN) + 1.50 x (nephrotoxic) + 3.10 x (obstructive) + 0.20 x eGFR + 0.36 x hemoglobin)]), where 1 for age category ≧65 years, 0 for <65 years; 1 for ischemic ATN, 0 for non-ischemic ATN; 1 for nephrotoxic, 0 for non-nephrotoxic; 1 for obstructive, 0 for non-obstructive; eGFR, and hemoglobin = observed values.

### Group comparisons of survival

**Fig 1:**
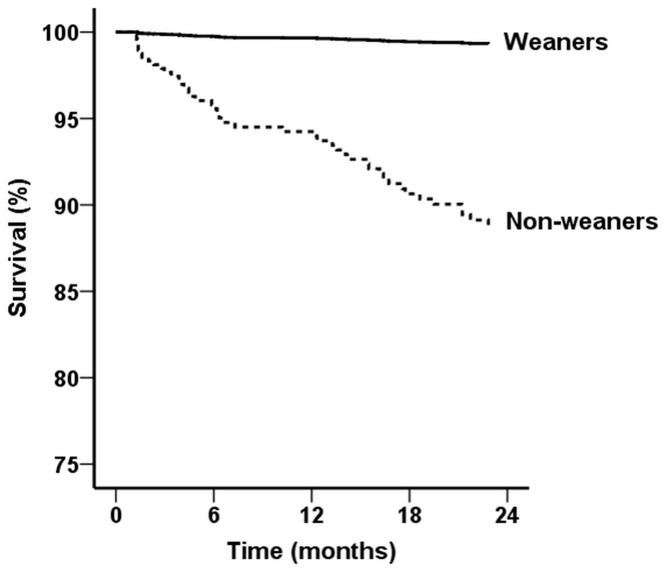
Cox proportional plots showing cumulative rates of overall surviva (adjusted hazard ratio 0.06; 95% confidence interval 0.01 to 0.35, p = 0.002) between the weaner and non-weaner groups.

During the 2-year follow-up, no differences in crude mortality rates were found between the weaners and non-weaners (5/36 [13.9%] vs. 59/385 [15.3%], p = 0.819 by Chi-squared test). Kaplan-Meier analysis also failed to show significant differences in time to overall survival between the 2 groups (p>0.05 by log-rank test). Then, to adjust for potential confounding due to case mix, we constructed Cox proportional hazard models to evaluate the effect of weaning off acute dialysis on time to overall mortality (Table 3). In model 1, only weaning from acute dialysis was assessed, and the univariate test showed a p value >0.05. In model 2, all potential variables except weaning from dialysis were entered. We observed that age ≧65 years, peripheral arterial occlusive disease, cancer, and eGFR showed a positive impact on early death, while serum albumin was associated with reduced risk of early death. In model 3, all potential predictors including weaning from dialysis were entered. We observed that successful weaning from dialysis showed a substantial negative association with early death. Finally, we adjusted the model for the propensity score for dialysis weaning and found that weaning off dialysis itself was an independent predictor of reduced early death (adjusted hazard ratio [HR] 0.06; 95% confidence interval [CI] 0.01 to 0.35, p = 0.002). The chance of early death after successful weaning from dialysis was decreased by 94% (model 4 and Fig 1). In addition to peripheral arterial occlusive disease, cancer, and serum albumin, we observed the presence of a history of hypertension showed a protective effect on early death, and a history of stroke exerted a negative impact on overall survival.

**Table 3:** Multivariate Cox proportional hazard models of independent predictors of all-cause mortality.

All-cause mortality	Model 1	Model 2*	Model 3*	Model 4**
Predicted probability	-	-	-	42.52 (4.77–379.25) p = 0.001
Weaners vs. non-weaners	0.85 (0.34–2.12) p = 0.732	-	0.20 (0.06–0.64) p = 0.007	0.06 (0.01–0.35) p = 0.002
Age category (≧65 vs. <65 yr)	-	3.35 (1.80–6.22) p<0.001	3.75 (2.01–7.00) p<0.001	-
Hypertension (yes vs. no)	-	0.66 (0.35–1.27) p = 0.214	0.74 (0.37–1.46) p = 0.383	0.47 (0.23–0.98) p = 0.045
CVA (yes vs. no)	-	1.24 (0.57–2.67) p = 0.585	1.44 (0.66–3.12) p = 0.358	3.00 (1.34–6.74) p = 0.008
PAOD (yes vs. no)	-	2.41 (1.11–5.26) p = 0.027	2.39 (1.10–5.19) p = 0.028	3.88 (1.70–8.83) p = 0.001
Cancer (yes vs. no)	-	3.37 (1.79–6.31) p<0.001	1.68 (1.06–2.67) p = 0.028	4.61 (2.19–9.69) p<0.001
eGFR (per mL/min/1.73 m^2^)	-	1.10 (1.02–1.19) p = 0.010	1.16 (1.07–1.26) p<0.001	-
Albumin (per g/dL)	-	0.61 (0.38–0.97) p = 0.037	0.56 (0.35–0.91) p = 0.018	0.55 (0.32–0.94) p = 0.030

Values are shown as hazard ratio (95% confidence interval). Abbreviations. CVA, cerebrovascular accident; eGFR, estimated glomerular filtration rate; PAOD, peripheral arterial occlusive disease.

* Adjusted for hemoglobin, and other comorbidities (diabetes mellitus, dyslipidemia, coronary artery disease, and congestive heart failure).

**Adjusted for dyslipidemia, coronary artery disease, and congestive heart failur*e*.

### Causes of death

Among the 64 deaths, 24 (37.5%) occurred between 31 and 90 days, 9 (14.1%) between 91 and 180 days, and 31 (48.4%) after 180 days. The most common causes of death were infection (34/64 [53.1%]), cancer (9/64 [14.1%]), and cardiovascular disease (8/64 [12.5%]). Twelve (35.3%) of the 34 infection-related deaths were considered CVC-associated, with 6 occurring in the first 3 months after acute dialysis.

### Readmission after hospital discharge

**Fig 2:**
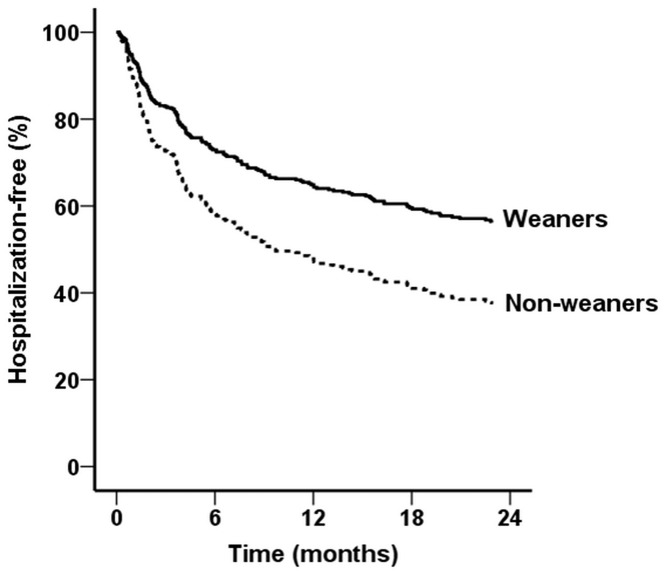
Cox proportional plots showing cumulative rates of overall rehospitalization (adjusted hazard ratio 0.59; 95% confidence interval 0.31 to 1.10, p = 0.095) between the weaner and non-weaner groups.

The effect of weaning off dialysis on time to readmission was analyzed with Cox proportional hazards models (Table 4). Of these, model 3 shows successful weaning from acute dialysis reduced the risk of hospital readmission by 43%. This benefit, however, became statistically insignificant after accounting for the propensity score in the final model (model 4 and Fig 2). Nevertheless, we observed the presence of a history of hypertension showed a protective effect on early readmission, while a history of coronary artery disease increased the likelihood of early readmission.

**Table 4:** Multivariate Cox proportional hazard models of independent predictors of overall rehospitalization.

Overall readmission	Model 1	Model 2^#^	Model 3^#^	Model 4^##^
Predicted probability	-	-	-	1.41 (0.44–4.59) p = 0.563
Weaners vs. non-weaners	0.79 (0.50–1.27) p = 0.331	-	0.57 (0.33–0.96) p = 0.036	0.59 (0.31–1.10) p = 0.095
Hypertension (yes vs. no)	-	0.67 (0.47–0.96) p = 0.027	0.67 (0.47–0.96) p = 0.028	0.59 (0.40–0.86) p = 0.006
Coronary artery disease (yes vs. no)	-	1.48 (1.05–2.09) p = 0.024	1.53 (1.09–2.16) p = 0.014	1.62 (1.11–2.36) p = 0.012

Values are shown as hazard ratio (95% confidence interval).

^#^Adjusted for age category, eGFR, albumin, hemoglobin, and other comorbidities (diabetes mellitus, dyslipidemia, congestive heart failure, cerebrovascular accident, PAOD, and cancer).

^##^Adjusted for albumin and other comorbidities (dyslipidemia, congestive heart failure, cerebrovascular accident, PAOD, and cancer).

## Discussion

This prospective observational study shows that a subset of patients with CKD who started dialysis acutely due to uremic urgency could be weaned off dialysis and remained dialysis-free over a period of 2 years. Importantly, successful weaning from acute dialysis was associated independently with reduced risk of early death, which highlights the needs for structured guidelines aiming for dialysis weaning in patients with existing CKD after urgent-start dialysis.

Most researches addressing the issue of discontinuing dialysis focus on withholding or foregoing life-sustaining dialysis [19,20], or withdrawal as cause of death [21]. By contrast, this study investigated therapeutic weaning from acute dialysis in patients with existing renal diseases who were not terminally ill or expected to commence long-term RRT in the short term. We identified several predictors associated with successful weaning from acute dialysis. Notably, higher predialysis eGFR and higher serum hemoglobin reflected lower chronicity and/or lesser severity of kidney disease, and the presence of which at entry might predict subsequent renal function recovery and dialysis weaning. On the other hand, exposure to ischemic injury or nephrotoxins, and presence of urinary obstruction are reversible etiologies of AKI [16], which explained why some patients could be removed from acute dialysis whereas others could not. Because the rate of successful weaning from acute dialysis is much lower in patients with AKI superimposed on CKD [8,11,22], closer surveillance is required for those patients who presented with the above-mentioned clinical features so that premature ESRD can be prevented. Interestingly, the older age group (≧65 years) showed a greater chance of weaning off dialysis. This age group displayed a lower level of serum creatinine at acute dialysis, which by itself was an independent predictor of dialysis weaning. Additionally, the older weaners showed a higher level of serum potassium than their younger counterpart, suggesting that some of these patients underwent acute dialysis due to unexpected hyperkalemia. The reversible nature of this complication might contribute in part to the higher weaning rate seen in the older age group. Conversely, this phenomenon was not evident in the non-weaner group (see S2 Table).

The mortality profile in this study was unique as we found that the overall mortality rate in the first 25-month following dialysis initiation was relatively low (1st month mortality 4.3%; 2nd to 25th month mortality 15.2%), compared with that collectively reported in incident ESRD patients in the west [23–25]. Although several possibilities could have explained the heightened mortality of dialysis patients in the United States [26], we surmise that ethnic variations in background mortality rates [27,28] may play a major part in the preferential survival of Asian patients with CKD both before and after commencement of long-term RRT [13,29,30]. Moreover, on top of the inherent survival advantage, we observed that weaning off acute dialytic therapy was independently associated with reduced risk of early death. Because the most common cause of death in this cohort was CVC-associated infection, as had been reported elsewhere in acute unplanned dialysis patients [24,31], it is important that every clinical effort should be made to wean off unanticipated dialysis if appropriate. And for the non-weaners who required long-term RRT, the general guidance of using a non-CVC dialysis access should be implemented so that inadvertently life-threatening bloodstream infections can be prevented.

Hypoalbuminemia is one of the serum markers of protein-energy wasting, and has been recognized as a major predictor of mortality in patients with CKD and ESRD [32]. Significantly, our study demonstrates that compared with serum albumin, weaning from acute dialysis showed a similar, if not stronger predictive power for reduced early death in dialysis-requiring patients with existing CKD. These results, together with other evidence [8–11], speak for the need of developing structured guidelines aiming for dialysis weaning in this particular patient group. In addition to weaning from dialysis, a history of hypertension, which occurred in 81% of the participants, was found to be an independent protector of early death and rehospitalization. Although such findings may seem unreasonable at first glance, it is our contention that a history of hypertension does not necessarily equate to a health risk. Instead, patients with existing hypertension may receive more regular clinic visits than those without, and any fluctuation of health conditions could have been detected and prompt therapies delivered when indicated. This supposition coincided conceptually with the observations that patients with preexisting kidney diseases displayed paradoxically improved outcomes from AKI [25,33]. In comparison, the presence of cerebrovascular disease, peripheral arterial disease, and cancer exerted a negative impact on overall survival. These observations coincided with previous investigations that showed poor clinical outcomes in patients with CKD or ESRD having similar underlying conditions or comorbidities [34–36].

Apart from a history of hypertension, this study identified the presence of coronary artery disease as an independent predictor of early readmission. This finding coincided with previous reports that showed negative impacts of acute myocardial infarction and coronary artery disease in patients with ESRD [37,38]. Collectively, these data emphasize the importance of providing more intense medical care for new-onset dialysis patients who exhibit certain specific cardiovascular diseases. Additionally, it is noteworthy that successful weaning from acute dialysis reduced the risk of hospital readmission by 43% (model 3). This benefit, however, disappeared after accounting for the propensity score in the final model. Nevertheless, the causal association between discontinuation of acute dialysis and reduction of hospital readmission appears logical. The weaners can be cared mostly at outpatient nephrology clinics, whereas the non-weaners may require imminent rehospitalizations for creation of dialysis access or management of access complications. The potential cost-savings as a result of reduced early readmissions after dialysis weaning may merit special attention in future research.

There are some limitations to this investigation. First, the study was conducted using daily practice decisions to wean patients from acute dialysis across three medical centers. Therefore, our findings could have been affected by confounders and may not be applied to other patient groups. However, we tried to minimize such sampling bias by constructing the propensity score using predicted probability in the regression equation, and the result showed that daily clinical efforts to remove acute dialysis was feasible even in patients with advanced CKD. Second, the long-term effect of weaning off acute dialysis cannot be adequately assessed due to the relatively short observation period. Nonetheless, the survival advantage seen in this study can provide short-term guidance on the safety of dialysis weaning for patients with known CKD following urgent-start dialysis. Finally, the study did not include initial urine output as a variable in the Cox analysis model due to incomplete recording of data at start of dialysis. But the importance of monitoring urine output in the decision making of weaning off or re-starting dialysis cannot be overemphasized for patients with CKD placed on acute dialysis [11,39].

In summary, this paper demonstrates that compared to patients with CKD who became ESRD after urgent-start dialysis, patients who could be successfully weaned from acute dialytic therapy were protected from the risk of early death over a 2-year follow-up period.

## Supporting Information

S1 Table Multivariate Cox proportional hazard models of independent predictors of (A) all-cause mortality and (B) overall rehospitalization in patients with stage 5 CKD. (DOC)

S2 Table Baseline demographic characteristics and biochemical data in (A) all participants, (B) the weaners, and (C) the non-weaners, stratified by age groups. (DOC)
