# Supplementary material for: Impact of Weaning from Acute Dialytic Therapy on Outcomes of Chronic Kidney Disease following Urgent-Start Dialysis
Source: PLoS One. 2015 Apr 9;10(4):e0123386. doi: 10.1371/journal.pone.0123386 (PMC4391852; doi:10.1371/journal.pone.0123386)
Supplement: S1 Table — (DOC) [file pone.0123386.s001.doc]

**S1 Table.** Multivariate Cox proportional hazard models of independent predictors of (A) all-cause mortality and (B) overall rehospitalization in patients with stage 5 CKD (n=383)

**(A)**

|  | Model 1 | Model 2* | Model 3* | Model 4** |
| --- | --- | --- | --- | --- |
| Predicted probability | - | - | - | 946.703 (50.268-17829.243)  p < 0.001 |
| Weaners vs. non-weaners | 0.619 (0.151-2.543)  p = 0.505 | - | 0.182 (0.024-1.376)  p = 0.099 | 0.148 (0.017-1.256)  p = 0.080 |
| Age category  (≧65 vs. < 65  year) | - | 2.928 (1.454-5.893)  p = 0.003 | 3.065 (1.521-6.173)  p = 0.002 | - |
| CVA (yes vs. no) | - |  |  | 3.158 (1.253-7.963)  p = 0.015 |
| PAOD (yes vs. no) | - |  |  | 2.937 (1.151-7.492)  p = 0.024 |
| Cancer (yes vs. no) | - | 4.548 (2.199-9.406)  p < 0.001 | 3.905 (1.856-8.216)  p < 0.001 | 5.340 (2.376-12.003)  p < 0.001 |
| eGFR (per mL/min/1.73 m^2^) | - | 1.327 (1.177-1.495)  p < 0.001 | 1.348 (1.195-1.520)  p < 0.001 | - |

Note. Values shown as hazard ratio (95% confidence interval).

Abbreviations. CVA, cerebrovascular accident; eGFR, estimated glomerular filtration rate; PAOD, peripheral arterial occlusive disease. *** Adjusted for albumin, hemoglobin, and other comorbidities (diabetes mellitus, hypertension, dyslipidemia, coronary artery disease, and congestive heart failure). ****Adjusted for albumin and other comorbidities (hypertension, dyslipidemia, coronary artery disease, and congestive heart failure).

**(B)**

|  | Model 1 | Model 2^#^ | Model 3^#^ | Model 4^##^ |
| --- | --- | --- | --- | --- |
| Predicted probability | - | - | - | 2.232 (0.297-16.756)  p = 0.435 |
| Weaners vs. non-weaners | 0.808 (0.451-1.445)  p = 0.472 | - | 0.664 (0.355-1.240)  p = 0.199 | 0.709 (0.371-1.355)  p = 0.298 |
| Hypertension  (yes vs. no) | - | 0.643 (0.442-0.936)  p = 0.021 | 0.646 (0.444-0.940)  p = 0.022 | 0.611 (0.409-0.911)  p = 0.016 |
| Coronary artery disease (yes vs. no) | - | 1.387 (0.961-2.004)  p = 0.081 | 1.403 (0.973-2.023)  p = 0.070 | 1.523 (1.027-2.260)  p = 0.037 |

Values are shown as hazard ratio (95% confidence interval).

^#^Adjusted for age category, eGFR, albumin, hemoglobin, and other comorbidities (diabetes mellitus, dyslipidemia, congestive heart failure, cerebrovascular accident, PAOD, and cancer). ^##^Adjusted for albumin and other comorbidities (dyslipidemia, congestive heart failure, cerebrovascular accident, PAOD, and cancer).
