# Supplementary material for: Impact of Weaning from Acute Dialytic Therapy on Outcomes of Chronic Kidney Disease following Urgent-Start Dialysis
Source: PLoS One. 2015 Apr 9;10(4):e0123386. doi: 10.1371/journal.pone.0123386 (PMC4391852; doi:10.1371/journal.pone.0123386)
Supplement: S2 Table — (DOC) [file pone.0123386.s002.doc]

***S2 Table. Baseline demographic characteristics and biochemical data in (A) all participants, (B) weaners, and (C) non-weaners, stratified by age groups.***

|  | All | ＜65 years | ≧65 years |
| --- | --- | --- | --- |
| No. of patients | 421 | 229 | 192 |
| Men, no. (%) | 240 (57) | 142 (62.0) | 98 (51.0)* |
| Mean age at entry (y) | 61.9±16.4 | 49.7±11.0 | 76.5±7.2* |
| Weaner, no. (%) | 36 (8.6) | 10 (4.4) | 26 (13.5)* |
| Primary renal disease, no. (%) | | | |
| Diabetes mellitus | 185 (44) | 101 (44.1) | 84 (43.8) |
| Glomerulonephritis | 93 (22) | 60 (26.2) | 33 (17.2)* |
| Others | 143 (34) | 68 (29.7) | 75 (39.1)* |
| Comorbidity at entry, no. (%) | | | |
| Diabetes mellitus | 213 (51) | 116 (50.7) | 97 (50.5) |
| Hypertension | 342 (81) | 184 (80.3) | 158 (82.3) |
| Dyslipidemia | 106 (25) | 56 (24.5) | 50 (26.0) |
| CAD | 69 (16) | 32 (14.0) | 37 (19.3) |
| CHF | 84 (20) | 36 (15.7) | 48 (25.0)* |
| VHD | 25 (6) | 6 (2.6) | 19 (9.9)* |
| Arrhythmia | 5 (1) | 2 (0.9) | 3 (1.6) |
| CVA | 39 (9) | 13 (5.7) | 26 (13.5)* |
| PAOD | 30 (7) | 12 (5.2) | 18 (9.4) |
| Cancer | 40 (10) | 13 (5.7) | 27 (14.1)* |
| Acute-on-chronic precipitating factor, no. (%) | | | |
| Ischemic ATN | 23 (6) | 8 (3.5) | 15 (7.8) |
| Nephrotoxic | 27 (6) | 19 (8.3) | 8 (4.2) |
| Cardiac | 157 (37) | 86 (37.6) | 71 (37.0) |
| Inflammatory/infectious | 20 (5) | 9 (3.9) | 11 (5.7) |
| Hepatic | 1 (0) | 0 (0) | 1 (0.5) |
| Obstructive | 12 (3) | 4 (1.7) | 8 (4.2) |
| Nil^+^ | 181 (43) | 103 (45.0) | 78 (40.6) |
| Renal sonography at entry | | | |
| Kidney size, cm | 9.5±1.5 | 9.5±1.5 | 9.5±1.4 |
| Baseline CKD stage, n (%) | | | |
| Stage 3B | 4 (1) | 0 (0) | 4 (2.1)* |
| Stage 4 | 34 (8) | 16 (7.0) | 18 (9.4) |
| Stage 5 | 383 (91) | 213 (93.0) | 170 (88.5) |
| Renal function at acute dialysis | | | |
| Blood urea nitrogen, mg/dL | 122.4±45.9 | 123.8±46.8 | 120.7±44.9 |
| Creatinine, mg/dL | 11.8±5.4 | 13.7±5.7 | 9.6±4.1* |
| eGFR (CKD-EPI),  mL/min/1.73 m^2^ | 4.6±2.5 | 4.2±2.4 | 5.1±2.6* |
| Laboratory data at acute dialysis | | | |
| Albumin, g/dL | 3.4±0.6 | 3.4±0.6 | 3.3±0.6 |
| Hemoglobin, g/dL | 8.6±1.8 | 8.4±1.8 | 8.8±1.7 |
| Potassium, mmol/L | 4.7±1.1 | 4.6±1.0 | 4.9±1.2* |
| Phosphorus, mg/dL | 6.9±2.3 | 7.4±2.5 | 6.2±2.0* |
| Calcium, mg/dL | 8.0±1.3 | 7.8±1.3 | 8.3±1.3* |

Abbreviations. ATN, acute tubular necrosis; CAD, coronary artery disease; CHF, congestive heart failure; CVA, cerebrovascular accident; eGFR, estimated glomerular filtration rate; PAOD, peripheral arterial occlusive disease; VHD, valvular heart disease. Values are expressed as number (percent) or mean ± standard deviation. *<0.05 vs. people＜65 years. ^+^Nil denotes absence of classic acute precipitating factors as defined in the PICARD study.

**(B)**

|  | Weaners | ＜65 years | ≧65 years |
| --- | --- | --- | --- |
| No. of patients | 36 | 10 | 26 |
| Men, no. (%) | 18 (50.0) | 5 (50.0) | 13 (50.0) |
| Mean age at entry (y) | 70.4±12.6 | 55.1±11.2 | 76.3±6.9* |
| Primary renal disease, no. (%) | | | |
| Diabetes mellitus | 15 (41.7) | 5 (50.0) | 10 (38.5) |
| Glomerulonephritis | 5 (13.9) | 1 (10.0) | 4 (15.4) |
| Others^+^ | 16 (44.4) | 4 (40.0) | 12 (46.2) |
| Comorbidity at entry, no. (%) | | | |
| Diabetes mellitus | 18 (50.0) | 7 (70.0) | 11 (42.3) |
| Hypertension | 28 (77.8) | 9 (90.0) | 19 (73.1) |
| Dyslipidemia | 14 (38.9) | 5 (50.0) | 9 (34.6) |
| CAD | 12 (33.3) | 5 (50.0) | 7 (26.9) |
| CHF | 11 (30.6) | 3 (30.0) | 8 (30.8) |
| VHD | 1 (2.8) | 0 (0) | 1 (3.8) |
| Arrhythmia | - | - | - |
| CVA | 6 (16.7) | 0 (0) | 6 (23.1) |
| PAOD | 3 (8.3) | 1 (10.0) | 2 (7.7) |
| Cancer | 6 (16.7) | 1 (10.0) | 5 (19.2) |
| Acute-on-chronic precipitating factor, no. (%) | | | |
| Ischemic ATN | 4 (11.1) | 0 (0) | 4 (15.4) |
| Nephrotoxic | 5 (13.9) | 2 (20.0) | 3 (11.5) |
| Cardiac | 10 (27.8) | 4 (40.0) | 6 (23.1) |
| Inflammatory/infectious | 2 (5.6) | 0 (0) | 2 (7.7) |
| Hepatic | 1 (2.8) | 0 (0) | 1 (3.8) |
| Obstructive | 7 (19.4) | 3 (30.0) | 4 (15.4) |
| Nil^+^ | 7 (19.4) | 1 (10.0) | 6 (23.1) |
| Renal sonography at entry | | | |
| Kidney size, cm | 10.3±1.3 | 10.4±1.1 | 10.3±1.4 |
| Baseline CKD stage, n (%) | | | |
| Stage 3B | 2 (5.6) | 0 (0) | 2 (7.7) |
| Stage 4 | 12 (33.3) | 3 (30.0) | 9 (34.6) |
| Stage 5 | 22 (61.1) | 7 (70.0) | 15 (57.7) |
| Renal function at acute dialysis | | | |
| Blood urea nitrogen, mg/dL | 106.5±55.6 | 90.2±43.0 | 112.8±59.3 |
| Creatinine, mg/dL | 7.4±2.8 | 8.3±2.9 | 7.0±2.7 |
| eGFR (CKD-EPI),  mL/min/1.73 m^2^ | 7.2±3.9 | 6.8±3.7 | 7.4±4.0 |
| Laboratory data at acute dialysis | | | |
| Albumin, g/dL | 3.3±0.6 | 3.5±0.8 | 3.2±0.6 |
| Hemoglobin, g/dL | 9.8±2.2 | 9.8±2.2 | 9.9±2.3 |
| Potassium, mmol/L | 5.3±1.7 | 4.3±1.4 | 5.7±1.7* |
| Phosphorus, mg/dL | 6.0±2.1 | 5.7±1.6 | 6.2±2.3 |
| Calcium, mg/dL | 8.5±1.6 | 8.8±1.4 | 8.4±1.7 |

Abbreviations. ATN, acute tubular necrosis; CAD, coronary artery disease; CHF, congestive heart failure; CVA, cerebrovascular accident; eGFR, estimated glomerular filtration rate; PAOD, peripheral arterial occlusive disease; VHD, valvular heart disease. Values are expressed as number (percent) or mean ± standard deviation. *<0.05 vs. people＜65 years. ^+^ Nil denotes absence of classic acute precipitating factors as defined in the PICARD study.

**(C)**

|  | Non-weaners | ＜65 year | ≧ 65 year |
| --- | --- | --- | --- |
| No. of patients | 385 | 219 | 166 |
| Men, no. (%) | 222 (57.7) | 137 (62.6) | 85 (51.2)* |
| Mean age at entry (y) | 61.1±16.5 | 49.5±11.0 | 76.5±7.3** |
| Primary renal disease, no. (%) | | | |
| Diabetes mellitus | 170 (44.2) | 96 (43.8) | 74 (44.6) |
| Glomerulonephritis | 88 (22.9) | 59 (26.9) | 29 (17.5)* |
| Others | 127 (33.0) | 64 (29.2) | 63 (38.0) |
| Comorbidity at entry, no. (%) | | | |
| Diabetes mellitus | 195 (50.6) | 109 (49.8) | 86 (51.8) |
| Hypertension | 314 (81.6) | 175 (79.9) | 139 (83.7) |
| Dyslipidemia | 92 (23.9) | 51 (23.3) | 41 (24.7) |
| CAD | 57 (14.8) | 27 (12.3) | 30 (18.1) |
| CHF | 73 (19.0) | 33 (15.1) | 40 (24.1)* |
| VHD | 24 (6.2) | 6 (2.7) | 18 (10.8)* |
| Arrhythmia | 5 (1.3) | 2 (0.9) | 3 (1.8) |
| CVA | 33 (8.6) | 13 (5.9) | 20 (12.0) |
| PAOD | 27 (7.0) | 11 (5.0) | 16 (9.6) |
| Cancer | 34 (8.8) | 12 (5.5) | 22 (13.3)* |
| Acute-on-chronic precipitating factor, no. (%) | | | |
| Ischemic ATN | 19 (4.9) | 8 (3.7) | 11 (6.6) |
| Nephrotoxic | 22 (5.7) | 17 (7.8) | 5 (3.0)* |
| Cardiac | 147 (38.2) | 82 (37.4) | 65 (39.2) |
| Inflammatory/infectious | 18 (4.7) | 9 (4.1) | 9 (5.4) |
| Obstructive | 5 (1.3) | 1 (0.5) | 4 (2.4) |
| Nil^+^ | 174 (45.2) | 102 (46.6) | 72 (43.4) |
| Renal sonography at entry | | | |
| Kidney size, cm | 9.4±1.5 | 9.4±1.5 | 9.4±1.4 |
| Baseline CKD stage, n (%) | | | |
| Stage 3B | 2 (0.5) | 0 (0) | 2 (1.2) |
| Stage 4 | 22 (5.7) | 13 (5.9) | 9 (5.4) |
| Stage 5 | 361 (93.8) | 206 (94.1) | 155 (93.4) |
| Renal function at acute dialysis | | | |
| Blood urea nitrogen, mg/dL | 123.9±44.7 | 125.3±46.5 | 121.9±42.3 |
| Creatinine, mg/dL | 12.2±5.4 | 14.0±5.7 | 10.0±4.1** |
| eGFR (CKD-EPI),  mL/min/1.73 m^2^ | 4.3±2.2 | 4.1±2.3 | 4.7±2.0** |
| Laboratory data at acute dialysis | | | |
| Albumin, g/dL | 3.4±0.6 | 3.4±0.6 | 3.4±0.6 |
| Hemoglobin, g/dL | 8.5±1.7 | 8.3±1.8 | 8.6±1.5 |
| Potassium, mmol/L | 4.6±1.0 | 4.6±0.9 | 4.7±1.0 |
| Phosphorus, mg/dL | 6.9±2.3 | 7.5±2.5 | 6.2±2.0** |
| Calcium, mg/dL | 8.0±1.3 | 7.8±1.2 | 8.3±1.3** |

Abbreviations. ATN, acute tubular necrosis; CAD, coronary artery disease; CHF, congestive heart failure; CVA, cerebrovascular accident; eGFR, estimated glomerular filtration rate; PAOD, peripheral arterial occlusive disease; VHD, valvular heart disease. Values are expressed as number (percent) or mean ± standard deviation. *<0.05 vs. people＜65 years. ^+^ Nil denotes absence of classic acute precipitating factors as defined in the PICARD study.
